# Transferring Learning from External to Internal Weights in Echo-State Networks with Sparse Connectivity

**DOI:** 10.1371/journal.pone.0037372

**Published:** 2012-05-24

**Authors:** David Sussillo, L.F. Abbott

**Affiliations:** 1 Department of Electrical Engineering, Stanford University, Stanford, California, United States of America; 2 Department of Neuroscience, Columbia University, New York, New York, United States of America; McMaster University, Canada

## Abstract

Modifying weights within a recurrent network to improve performance on a task has proven to be difficult. Echo-state networks in which modification is restricted to the weights of connections onto network outputs provide an easier alternative, but at the expense of modifying the typically sparse architecture of the network by including feedback from the output back into the network. We derive methods for using the values of the output weights from a trained echo-state network to set recurrent weights within the network. The result of this “transfer of learning” is a recurrent network that performs the task without requiring the output feedback present in the original network. We also discuss a hybrid version in which online learning is applied to both output and recurrent weights. Both approaches provide efficient ways of training recurrent networks to perform complex tasks. Through an analysis of the conditions required to make transfer of learning work, we define the concept of a “self-sensing” network state, and we compare and contrast this with compressed sensing.

## Introduction

Training a network typically involves making adjustments to its parameters to implement a transformation or map between the network’s input and its output, or to generate a temporally varying output of a specified form. Training in such a network could consist of modifying some or all of its weights. Learning schemes that modify the recurrent weights are notoriously difficult to implement [1]–[2] (although see [3]). To avoid these difficulties, Maass and collaborators [4] and Jaeger [5] suggested limiting synaptic modification during learning to the output weights, leaving the recurrent weights unchanged. This scheme greatly simplifies learning, but is limited because it does not allow the dynamics of the recurrent network to be modified. Jaeger and Haas [6] proposed a clever compromise in which modification is restricted to the output weights, but a feedback loop carries the output back into the network. By permitting the output to affect the network, this scheme modifies the intrinsic dynamics of the network. FORCE learning was developed as an efficient algorithm for implementing this approach with the benefits of creating stable networks and enabling the networks to operate in a more versatile regime [7].

**Figure 1:**
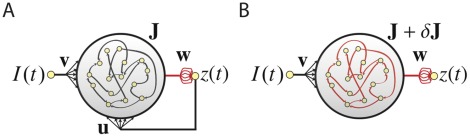
The two recurrent network architectures being considered. The nets are shown with non-modifiable connections shown in black and modifiable connections in red. Both networks receives input 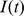, contain units that interact through a sparse weight matrix 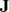, and produce an output 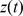, obtained by summing activity from the entire network weighted by the modifiable components of the vector 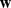. (A) The output unit sends feedback to all of the network units through connections of fixed weight 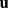. Learning affects only the output weights 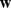. (B) The same network as in A, but without output feedback. Learning takes place both in the network through the modification 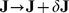, to implement the effect of the feedback loop, and at the output weights 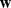, to correctly learn 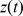.

While the echo-state approach greatly expands the capabilities for performing complex tasks [6] [8] [7], this capacity comes at the price of altering the architecture of the network through the addition of the extra feedback loop (Figure 1A), effectively creating an all-to-all coupled network. In neuroscience applications in particular, the original connectivity of the network is typically restricted to match anatomical constraints such as sparseness, but the additional feedback loop may violate these constraints by being non-sparse or excessively strong, and thus may be biologically implausible. This raises an interesting question: Can we train a network without feedback (Figure 1B) to perform the same task as a network with feedback (Figure 1A), using the same output weights, by modifying the internal, recurrent connections?

The answer is yes, and previously [7] we described how the online FORCE learning rule could be applied simultaneously to recurrent and output weights in the absence of an output-to-network feedback loop (Figure 1B). We now expand this result in three ways. First, we develop batch equations for transferring learning achieved using a feedback network with online FORCE learning to the recurrent connections of a network without feedback. The reason for this two-step approach is that it speeds up the learning process considerably. Second, we use results from this first approach to more rigorously derive the online learning rule for training recurrent weights that we proposed previously [7]. Third, we introduce the concept of a self-sensing network state, and use it to explore the range of network parameters under which internal FORCE learning works.

There has been parallel work in studying methods for internalizing the effects of trained feedback loops into a recurrent pool. These studies focused on control against input perturbations [9]–[10], regularization [11] and prediction [12]. The principle issue that we study in this manuscript is motivated from a computational neuroscience perspective: what are the conditions under which transfer of external feedback loops to the recurrent network will be successful, while preserving sparse connectivity. Maintenance of sparsity requires us to work within a random sampling framework. Our focus on respecting locality and sparseness constraints increases the biological relevance of our results and leads to a network learning rule that only requires a single, global error signal to be conveyed to network units.

## Results

Our network model (Figure 1) is described by an 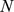-dimensional vector of activation variables, 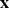, and a vector of corresponding “firing rates”, 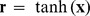
(other nonlinearities, including non-negative functions, can be used as well). The equation governing the dynamics of the activation vector for the network of Figure 1B is of the standard form
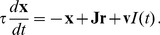
(1)The time constant 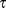 has the sole effect of setting the time scale for all of our results. For example, doubling 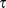 while making no other parameter changes would make the outputs we report evolve twice as slowly. The 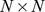 matrix 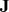 describes the weights of the recurrent connections of the network, and we take it to be randomly sparse, meaning that only 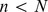 randomly chosen elements are non-zero in each of its rows. The non-zero elements of 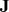 are initially drawn independently from a Gaussian distribution with zero mean and variance 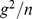. The parameter 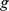, when it is greater than 1, determines the amplitude and frequency content of the chaotic fluctuations in the activity of the network units. In order for FORCE learning to work, 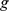 must be small enough so that feedback from the output into the network can produce a transition to a non-chaotic state (see below and Sussillo and Abbott, 2009). The scalar input to the network, 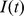, is fed in through the vector of weights 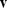 with elements drawn independently and uniformly over the range 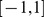. Thus, up to the scale factors 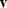, every unit in the network receives the same input.

The output of the network, 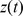, is constructed from a linear sum of the activities of the network units, described by the vector 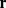, multiplied by a vector of output weights 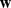 [13] [4]–[5],
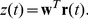
(2)Training in such a network could, in principal, consist of modifying some or all of the weights 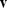, 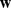 or 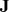. In practice, we restrict weight modification to either 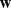 alone (Figure 1A), or 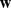 and 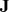 (Figure 1B). Increasing the number of inputs or outputs introduces no real difficulties, so we treat the simplest case of one input and one output.

The idea introduced by Jaeger and Haas [6], which allows learning to be restricted solely to the output weights 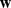, is to change equation 1 for the network of Figure 1B to.
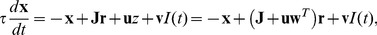
(3)for the network of Figure 1A. The components of 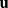 are typically drawn independently and uniformly over the range 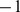 to 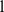 and are not changed by the learning procedure. As indicated by the second equality in equation 3, the effective connectivity matrix of the network with the feedback loop in place is 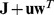. This changes when 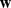 is modified, even though 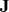, 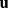 and 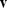 remained fixed. This is what provides the dynamic flexibility for this form of learning.

The problem we are trying to solve is to duplicate the effects of the feedback loop in the network of Figure 1A by making the modification 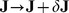 in the network of Figure 1B. A comparison of equations 1 and 3 would appear to provide an obvious solution; simply set 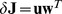. In other words, the network without output feedback is equivalent to the network with feedback if the rank-one matrix 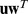 is added to 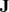. The problem with this solution is that the replacement 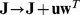 typically violates the sparseness constraint on 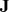. Even if both 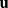 and 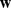 are sparse, it is unlikely that the outer product 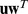 will satisfy the specific sparseness conditions imposed on 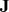. This is the real problem we consider; duplicating the effect of the addition of a rank-one matrix to the recurrent connectivity by a modification of higher rank that respects the sparseness of the network.

### Review of the FORCE Learning Rule

Because the FORCE learning algorithm provides the motivation for our work, we briefly review how it works. More details can be found in [7]. The FORCE learning rule is a supervised learning procedure, based on the recursive least squares algorithm (see [14]), that is designed to stabilize the complex and potentially chaotic dynamics of recurrent networks by making very fast weight changes with strong feedback. We describe two versions of FORCE learning, one applied solely to the output weights of a network with the architecture shown in Figure 1A, and the other applied to both the recurrent and output weights of a network of the form shown in Figure 1B. In both cases, learning is controlled by an error signal,
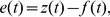
(4)which is the difference between the actual network output, 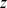, and the desired or target output, 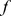.

For the architecture of Figure 1A, learning consists of modifications of the output weights made at time intervals 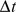 and defined by
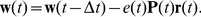
(5)

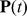
 is a running estimate of the inverse of the network correlation matrix,
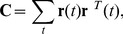
(6)where the sum over 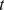 refers to a sum over samples of 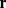 taken at different times. FORCE learning is based on a related matrix 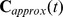 that is initially set proportional to the identity matrix, 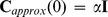. At each learning interval, 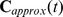 is updated with a sample of 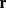, so that 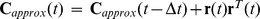. As 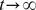, 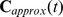 approaches the correlation matrix 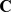 defined in equation 6 (more precisely, they approach each other if normalized by the number of samples). At each time step, 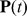 is the inverse of 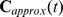, however it does not have to be determined by computing a matrix inverse. Instead, it can be computed recursively using the update rule, which is derived from the Woodbury matrix identity [14],
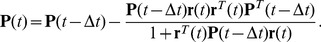
(7)Equations 5 and 7 define FORCE learning applied to 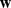. The factor 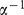 acts both as the initial learning rate and as a regularizer for the recurrsive matrix inversion being performed. By setting 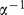 to a large value, the learning rule is able to drive the network out of the chaotic regime by feeding back a close approximation of the target signal 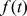 through the feedback weights 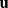 [7].

As learning progresses, the matrix P acts as a set of 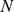 learning rates with a 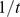 annealing schedule. This is seen most clearly by shifting to a basis in which P is diagonal. Provided that learning has progressed long enough for P to have converged to the inverse correlation matrix of 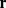, the diagonal basis is achieved by projecting 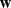 and 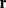 onto principal component (PC) vectors of 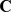. In this basis, the learning rate, 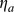, for the component of 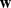 aligned with PC vector 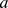 after 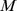 weight updates is 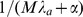, where 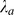 is the corresponding PC eigenvalue. This rate divides the learning process into two phases. The first is an early control phase when 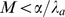 and 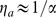 and the major role of weight modification is virtual teacher forcing, that is to keep the output close to 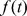 and drive the network out of the chaotic regime. The second phase begins when 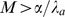 and 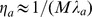, and now the goal of weight modification is traditional learning, i.e. to find a static set of weights that makes 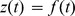. Components of 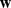 with large eigenvalues quickly enter the learning phase, whereas those with small eigenvalues spend more time in the control phase. Controlling the components with small eigenvalues allows weight projections in dimensions with large eigenvalues to be learned despite the initial chaotic state of the network. At all times during learning, the network is driven through 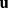 with a signal that is approximately equal to 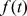, thus the name FORCE Learning - First Order Reduced and Controlled Error Learning.

FORCE learning was also proposed as a method for inducing a network without feedback (Figure 1B) to perform a task by simultaneously modifying 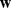 and 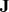. In this formulation, equations 5 and 7 are applied to the actual output unit and, in addition, to each unit of the network, which is treated as if it were providing the output itself. In other words, equations 5 and 7 are applied to every unit of the network, including the output, all using the same error signal defined by equation 4. The only difference is that the modification in equation 5 for network unit 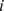 is applied to the vector of weights 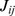 for all 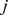 for which 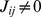 rather than 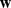, and the values of 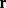 used in equations 5 and 7 are restricted to those values providing input to unit 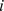. Details of this procedure are provided in [7] and, in addition, this “in-network” algorithm is re-derived in a later section below. The idea of treating a network unit as if it were an output is also a recurring theme in the following sections.

### Learning in Sparse Networks

Because sparseness constraints are essential to the problem we are considering, it is useful to make the sparseness of the network explicit in our formalism. To do this, we change the notation for 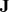. Each row of 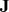 has only 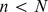 non-zero elements. We collect all the non-zero elements in row 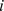 of the matrix 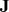 into an 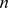-dimensional column vector 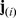. In addition, for each unit (unit 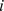 in this case) we introduce an 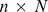 matrix 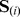 that is all zeros except for a single 1 in each row, with the location of the 1 in the 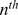 row indicating the identity of the 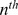 non-zero connection in 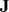. Using this notation, equation 1 for unit 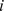 can be rewritten as
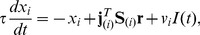
(8)a notation that, as stated, explicitly identifies and labels the sparse connections. This is only a change of notation, the set of equations 8 for 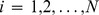 is completely equivalent to equation 1. However, in this notation, the sparseness constraint on 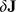 is easy to implement; we can modify the 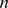-dimensional vectors 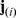, for 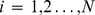 by 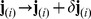 with no restrictions on the vectors 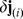.

According to equation 8, the modification 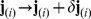 induces an additional input to unit 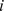 given by 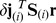. This will duplicate the effect of the feedback term in equation 3, if we can choose 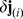 such that
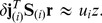
(9)The goal of learning in a sparse network is to make this correspondence as accurate as possible for each unit (exact equality may be unattainable). By doing this, the total input to unit 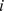 in the network of Figure 1B is whatever it receives through its original recurrent connections plus the contribution from changing these connections, 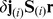, which is now as equal as possible to the input provided by the feedback loop, 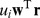, in the network with feedback (Figure 1A). In this way, a network without an output feedback loop operates as if the feedback were present.

#### Equivalence of training a sparse unit and a sparse output

Equation 9, which is our condition on the change 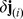 of the sparse connections for unit 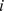, is similar in form to equation 2 that defines the network output. To make this correspondence clearer we write.
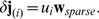
(10)Each unit of the network has its own vector 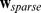 if this equation is applied to all network units, so 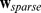 should really have an identifying index 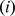 similar to the subscript on 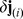. However, because each network unit is statistically equivalent in a randomly connected network with fixed sparseness per unit, we can restrict our discussion, at this point, to a single unit and thus a single vector 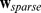. This allows us to drop the identifier 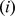, which avoids excessive indexing. Similarly, we will temporarily drop the 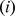 index on 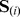, simply calling it 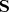. We return to discussing the full ensemble of network units and re-introduce the index 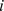 in a following section.

From equation 9, we can define the quantity.
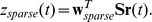
(11)Satisfying equation 9 as nearly as possible then amounts to making 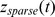 as close as possible to 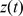. Comparing equation 2 and 11 shows that, although 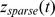 arises from our consideration of the recurrent inputs to a network unit, it is completely equivalent to an output extracted from the network, just as 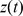 is extracted, except that there is a sparseness constraint on the output weights. Therefore, the problem we now analyze, which is how can 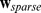 be chosen to minimize the difference between 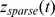 and 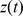, is equivalent to examining how accurately a sparsely connected output can reproduce the signal coming from a fully connected output. In order for our results to apply more generally, we allow the number of connections to this hypothetical sparse unit, which is the dimension of 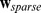 to be any integer 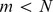, although for the network application we started with and will come back to, 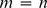.

We optimize the match between 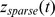 and 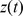 by minimizing 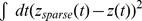. Solving this least-squares problem gives
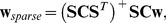
(12)with 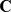 defined by equation 6. The superscript 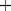 indicates a pseudoinverse, which is needed here because 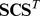 may not be invertible. The matrix being pseudoinverted in equation 12 is not the full correlation matrix, but rather 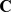 restricted to the 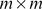 elements corresponding to correlations between units connected to the sparse output or, equivalently, the network unit that we are considering. This pseudoinverse matrix multiplies (with the sum in the matrix product restricted by 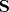 to sparse terms) the correlation matrix times the full weight vector. Note that if 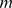 is equal to 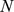 and the connections are labeled in a sensible way, 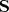 is the identity matrix and equation 12 reduces to 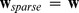. This recovers the trivial solution for modifying the network connections implied by the second equality in equation 3. We now study the non-trivial case, when 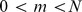.

For what follows, it is useful to express equation 12 in the basis of principal component vectors. To do this, we express 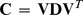, where 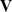 is the 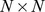 matrix constructed by arranging the eigenvectors of 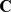 into columns, and 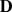 is the diagonal matrix of eigenvalues of 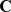 (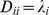, the i^th^ eigenvalue of 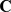). These eigenvectors are the principal component (PC) vectors. We arrange the diagonal elements of 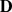 and the columns of 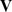 so that they are in decreasing order of PC eigenvalue. Using this basis, we introduce.
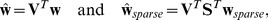
(13)where the hats denote vectors described in the PC basis. In this basis, equation 12 becomes


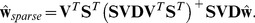
(14)

### The Dimension of Network Activity

Equation 11 corresponds to a sparsely connected unit with 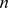 input connections attempting to extract the same signal 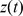 from a network as the fully connected output. For this to be done, it must be possible to access the full dynamics of 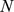 network units from a sampling of only 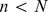 of them. The degree of accuracy of the approximate equality in equation 9 that can be achieved depends critically on the dimension of the activity of the network.

At any instant of time, the activity of an 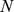-unit network is described by a point in an 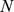-dimensional space, one dimension for each unit. Over time, the network state traverses a trajectory across this space. The dimension of network activity is defined as the minimum number of dimensions into which this trajectory, over the duration of the task being considered, can be embedded. If this can only be done to a finite degree of accuracy, we refer to the *effective dimension* of the network. The key feature of the networks we consider is that the effective dimension of the activity is typically less than, and often much less than, 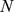.

For most networks performing tasks that involve inputs and parameters with reasonable values, the PC eigenvalues fall rapidly, typically exponentially [15] [7] [16]. Thus, we can write 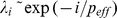, where 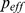 acts as an effective dimension of the network activity. If 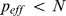, this raises the possibility that only 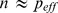 rates can provide access to all the information needed to reconstruct the activity of the entire network. Therefore, we ask how many randomly chosen rates are required to sample the meaningful dimensions of network activity? In addressing this question, we first consider the idealized case when 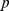 PC eigenvalues are nonzero and 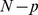 are identically zero. We then consider an exponentially decaying eigenvalue spectrum.

### Accuracy of Sparse Readout

For the idealized case where the activity of the network is strictly 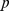-dimensional, we define 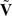 as the 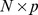 matrix obtained by keeping only the first 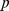 columns of 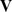 and similarly 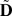 is the 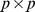 diagonal matrix obtained by keeping only the nonzero diagonal elements of 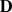. When 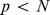, we can replace 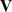 and 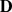 in equation 14 by 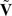 and 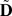, and ignore the components of 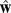 beyond the first 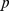. Equation 14 then becomes
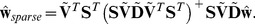
(15)The matrix 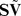 has dimension 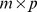 and thus is not invertible if 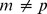. However, provided that the 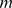 rows of 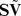 span 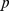 dimensions (see the final section before the Discussion), we have
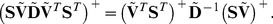
(16)Furthermore, if 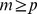, 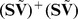 is equal to the identity matrix (although 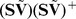 is not). As a result,
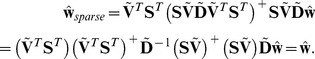
(17)Therefore, 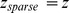, and we find that a sparse output or a network unit with 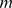 connections can reproduce the full output perfectly if 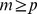 and 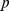, the dimension of the network activity, is less than 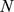.

When the PC eigenvalues fall off exponentially with effective dimension 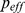, sparse reconstruction of a full network output is not perfect, but it can be extremely accurate. The error in approximating a fully connected output with a sparse output depends, of course, on the nature of the full output, which is determined by 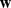. To estimate the error, and to compute it in network simulations, we assume that the components of 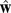 are chosen independently from a Gaussian distribution with zero mean and variance 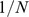. This is in some sense a worst case because, in applications involving a specific task, we expect that the components of 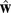 corresponding to PC vectors with large eigenvalues will dominate. Thus, the accuracy of sparse outputs in specific tasks (where 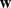 is trained) is likely to be better than our error results with generic output weights.

The error we wish to compute is 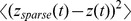. As a standard against which to measure this error, we introduce another, more common way of approximating a full output using only 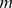 terms; simply by using the first 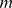 components of 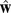 (in the PC basis) to construct an approximate output that we denote as 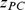. The error 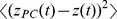 is easy to estimate, because this approximation matches the first 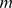 PCs exactly and sets the rest to zero. The error coming from the 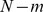 missing components is
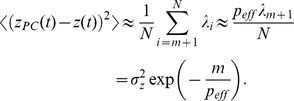
(18)


Here, the factor of 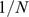 is the expected value of the square of each component of 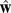, and the sum over eigenvalues is the sum of the expected values of the squared amplitudes of the modes with 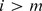. The second approximate equality follows from setting 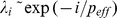, doing the geometric sum, ignoring a term 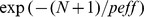, and using the approximation 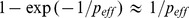. In the final equality of equation 18, we have normalized the error by the output variance 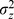.
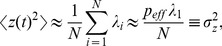
(19)using the same set of results and approximations as for equation 18. In this context, the squared error of the approximation is expressed as the fraction of the output variance that is missing.

**Figure 2:**
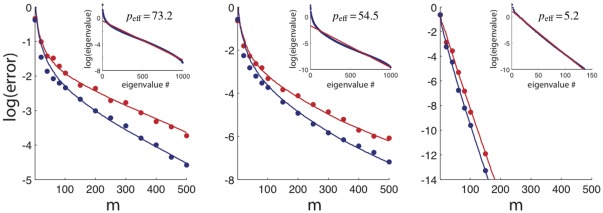
Comparison of network simulations and analytical results. The network simulations (filled circles) and analytic results (solid lines) for sparse (red) and PC (blue) reconstruction errors as a function of 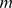 for different 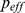 values. The “error” here is either 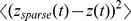 (red points and curve) or 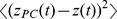 (blue points and curve). The input was 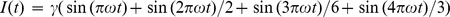 with 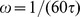 and 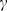 = 0, 0.4, 0.6 in the three panels, from left to right. The value of 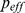 was adjusted by changing 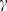. Inserts show the PC eigenvalues (blue) and the exponential fits to them (red), using the value of 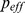 indicated. Logarithms are base 10.

We expect the error for 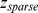 to be larger than 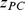 because 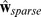 does not perfectly match the first 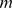 components of 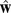, nor does it approximate the remaining components as zero. We extracted a good fit to the error for a sparse output with 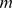 connections when the effective network dimension is 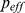 by studying a large number of numerical experiments and network simulations (for examples, see Figure 2). We found that this error is well-approximated by.
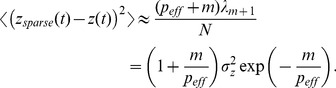
(20)


The difference between the accuracy of the output formed by 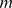 random samplings of 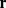 and that constructed by a PC analysis is the factor 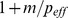 in equation 20 grows with 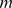, but it multiplies a term that decays exponentially as 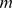 increases. Thus, using 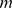 randomly selected inputs is almost as good as using an optimal PC approximation with 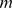 modes. The latter requires full knowledge of the eigenvectors and the locations of the meaningful PC dimensions, whereas the former relies only on random sampling.

To illustrate the accuracy of these results, we constructed a network with 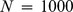, 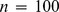, 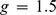 and 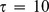 ms, and injected a time-dependent input with variable amplitude. Changing the amplitude of the input allowed us to modulate 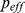, which is a decreasing function of input amplitude [17]. The readout weights, 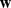, were selected randomly so that all modes of the network were sampled. There is good agreement between the results of the network simulation for the error in 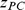 (filled blue circles) and equation 18 (blue curve), and the error in 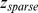 (filled red circles) and our estimate, equation 20 (red curve). Both equations fit the simulation data over a wide range of 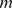 and 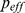 values.

### Transfer of Learning from a Feedback to a Non-Feedback Network

We now return to the full problem of adjusting the recurrent weights for every unit in a network in order to reproduce the effects of an output feedback loop. This merely involves extending the previous results from a single unit to all the units. In other words, we combine equations 10 and 12 to obtain an equation determining 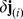 for all 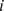 values,
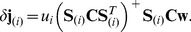
(21)Note that we have restored the 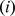 indexing that identifies the sparseness matrices for each unit. If these adjustments satisfy equation 9 to a sufficient degree of accuracy, a network of the form shown in Figure 1B, with the synaptic modification and output weights 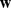 should have virtually identical activity to a network with unmodified recurrent connections, the same output weights, and feedback from the output back to the network (Figure 1A). We discuss the conditions required for this to happen in the final section before the Discussion.

**Figure 3:**
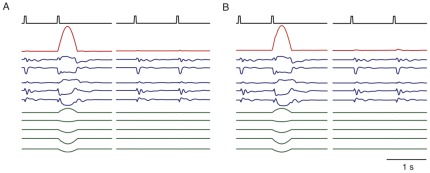
An example input-output task implemented in a network with feedback (A) and then transferred to a network without feedback using **equation 21** **.** The upper row shows the input to the network, consisting of two pulses separate by less than 1 s (left columns of A and B) or more than 1 s (right columns of A and B). The red traces show the output of the two networks correctly responding only to the input pulses separated by less than 1 s. The blue traces show 5 sample network units. The green traces show 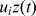 in A and 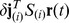 in B for the five sample units. The similarity in these traces shows that the transfer was successful at getting the recurrent input in B to approximate well the feedback input in A for each unit.

An example of a network constructed using equation 21 is shown in Figure 3. First, a network (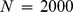, 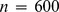, 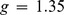, 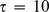 ms) with output feedback was trained with online FORCE learning to generate an output pulse after receiving two brief input pulses, but only if these pulses were separated by less than 1 second (Figure 3A, left column). When presented with input pulses separated by more than 1 second, the network was trained not to produce an output pulse (Figure 3A, right column). The input pairs were always either less than 975 ms or more than 1025 ms apart to avoid ambiguous intervals extremely close to 1 s. The learning was then batch transferred to the recurrent connections using equations 21, and the output feedback to the network was removed. After this transfer of learning to the sparse recurrent weights, the network performed almost exactly as it did in the original configuration (Figure 3B). Over 940 trials, the original feedback network performed perfectly on this task, and the network with no feedback but learning transferred to its recurrent connections performed with 98.8% accuracy. The green traces in Figure 3 show that 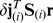 matches 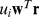 quite accurately.

#### Relation to simultaneous online learning of 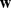 and 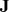


The previous section described a batch procedure for transferring learning from output weights to recurrent connections. It is also possible to implement this algorithm as an online process. To do this, rather than duplicating the complete effects of feedback with output weight vector 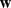 by making a batch modification 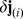, we can make a series of modifications 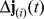 at each learning time step that duplicate the effects of a sequence of weight changes 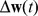. We could accomplish this simply by applying equation 21 at each learning time step, replacing the factor of 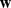 with 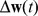. However, this would assume that we knew the correlation matrix 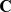, whereas FORCE learning, as described earlier, constructs this matrix (actually a diagonally loaded version of its inverse) recursively. Therefore, the correct procedure is to replace the factors of 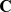 in equation 21, when it is applied at time 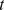, by 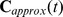. Similarly, the matrix 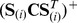 in equation 21 is replaced by a running estimate, updated by an equation analogous to equation 7,
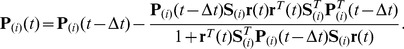
(22)There is no problem with doing the inverse (rather than pseudoinverse) here because, as a consequence of setting 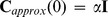, 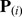 is diagonally loaded.

The recursive learning rule for modifying 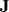 in concert with the modification of the output weights (equation 5) is then 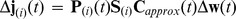. Using equation 5 to specify 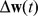, we find that 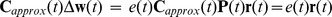 because 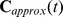 and 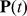 are inverses of each other. Thus,
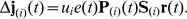
(23)


The factor of 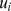 is needed if these modifications are designed to match those of a specific output feedback loop that uses 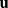 as its input weights. If all that is required is to generate a network without a feedback loop (Figure 1B) that does a desired task, any non-singular set of 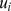 values can be chosen, for example 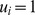 for all 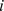. Equation 23 is equivalent to the learning rule proposed previously when this particular choice of 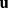 is made [7]. Note that all recurrent units and outputs are changing their weights through exactly the same functional form using only the global error and information that is local to each unit. Please see Appendix S1 in the supplemental materials for a derivation of these equations using index notation, which may be more helpful for implementation on a computer.

### Self-Sensing Networks and Compressed Sensing

We can now state the condition for successful transfer of learning between the networks of Figures 1A and 1B. This condition defines our term *self-sensing*. We require that, for each unit in the network, an appropriate modification of its sparse set of input weights allows the unit to approximate any function that can be extracted from the activity of the network by a linear readout with full connectivity. In other words, with an appropriate choice of 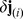, 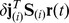 can approximate any readout, 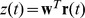, for all 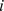 from 1 to 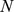.

Self-sensing and our analysis of it have relationships to the field of compressed sensing [18]–[19]. Both consider the possibility of obtaining complete or effectively complete knowledge of a large system of size 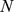 from 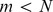 (and often 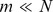) random samples. Self-sensing, as we have defined it, refers to the accuracy of outputs derived from random sparse samples of network activity. Compressed sensing refers to complete reconstruction of a sparse data set from random sampling. The problem in compressed sensing is that the data can arise from a large or even infinite set of different low-dimensional bases, and the reconstruction procedure is not provided with knowledge about which basis is being used. In self-sensing, the sparse basis is given by PCA, but the problem is that a sparsely connected unit cannot perform PCA on the full activity of the network. No matter what computational machinery is available to a unit for computing PCs, it cannot find the high variance PC vectors due to a lack of information. In a parallel and distributed setting, the only strategy for a unit with sparse inputs to determine what a network is doing is through random sampling. The general requirements for both self- and compressed sensing arise from their dependence on random sampling. The conditions for both are similar because it is as difficult to randomly sample sparsely from a single, unknown low-dimensional space as it is to sample from a sparse one when the low-dimensional state is unknown.

Our approach to constructing weights for sparse readouts is to start with the matrix of PC eigenvectors 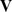, keep only the 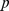 relevant vectors giving 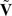, and then randomly sample 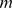 components from each of these vector, giving the matrix 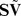 (e.g. see equation 14). Random sampling of this form will fail, that is generate zero vectors, if any of the eigenvectors of 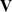 are aligned with specific units or if the 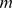 columns of 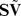 fail to span 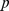 dimensions. These requirements for a self-sensing network correspond to the general concepts of incoherence and isotropy in the compressive sensing literature [19]. Put into our language, incoherence requires that the important PC eigenvectors not be concentrated onto a small number of units. If they were, it is likely that our random sparse sampling would miss these units and thus would have no access to essential PC directions. Isotropy requires that, over the distribution of random samples (all 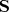), the columns of 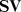 are equally likely to point in all directions. This corresponds to our requirement that the 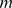 rows of the matrix 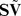 span 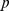 dimensions.

To be more specific, a random sampling of the network will fail to sample all of the modes of the network if some of the modes are created by single units. This problem can be eliminated by imposing an incoherence condition that the maximum element of 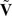 be of order 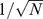 [18], which ensures that 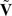 is rotated well away from the single-unit basis (the basis in which each unit corresponds to a single dimension). We require this condition, but it is almost certain to be satisfied in the networks we consider. One reason for this is that the connectivity described by 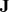 is random, and no single or small set of units in the networks we consider are decoupled from the rest of the network. Further, random connections induce correlations between units, and these correlations almost always ensure that the eigenvector basis is rotated away from the single-unit basis. Even if such an aligned eigenvector existed, the loss in reconstruction accuracy would likely be small because the 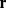 variables defining the correlation matrix are bounded. This implies that it is unlikely that an aligned mode would be among those with the largest eigenvalues because eigenvectors involving all of the units can construct larger total variances.

**Figure 4:**
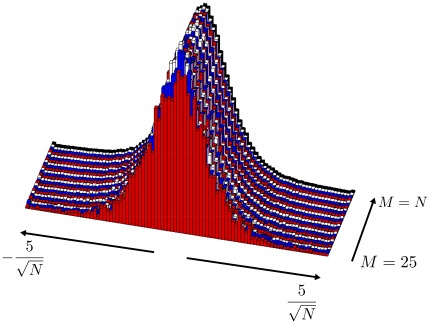
The distribution of the elements of 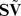, for equally spaced values of 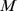. The eigenvectors 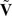 for a correlation matrix from simulations similar to those in figure 2 used to demonstrate the approximately Gaussian distribution for the elements of 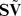. The red distribution in the front is for 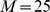, and the black distribution in the back is for 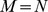, with intermediate layers corresponding to intermediate values. The 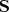 matrix was randomly initialized for each value of 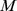.

We now address the isotropy condition, which in our application means that the 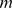 columns of 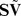 span 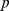 dimensions, as was required to prove that sparse reconstruction is exact if 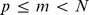 (equation 17). The columns of the full eigenvector matrix 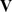 are constrained to be orthogonal and so, of course, they isotropically sample the network space. However, if 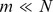, the column vectors of 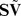 are no longer orthogonal. We make the assumption that, in this limit, the elements selected by the random matrix 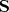 can be treated as independent random Gaussian variables. Studies of 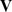 matrices extracted from network activity and randomly sparsified support this assumption (Figure 4). If 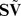 is a random Gaussian variable, the 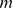 columns of 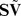 are unbiased and isotropically sample the relevant 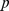 dimensional space.

In networks with a strictly bounded dimensionality of 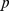, self-sensing requires 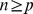. In networks with exponentially falling PC eigenvalues, self-sensing should be realized with an accuracy given by equation 20 if 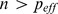. The effective dimensionality is affected by the inputs to a network, which reduce 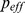 for increasing input amplitude, and the variance of the elements of 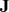 (controlled by 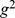), which increases 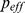 for increasing 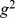. In response to an input [17] or during performance of a task, 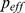 drops dramatically and is likely to be determined by the nature of the task rather than by 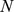. The crucial interplay is then between the scale of the input and the variance of 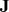, controlled by 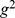. The self-sensing state should be achievable in many applications where the networks are either input driven or are pattern generators that are effectively input driven due to the output feeding back.

## Discussion

We have presented both batch and online versions of learning within a recurrent network. The fastest way to train a recurrent network without feedback is first to train a network with feedback and then to transfer the learning to the recurrent weights using equation 21. This will work if the network is in what we have defined as a self-sensing state.

An interesting feature of the online learning we have derived is that equation 23, specifying how a unit internal to the network should change its input weights, and equation 5 determining the weight changes for the network output, are entirely equivalent. Both involve running estimates of the inverse correlation matrix of the relevant inputs (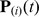 for network unit 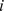 and 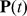 for the output) multiplying the firing rates of those inputs (either 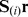 or 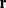). Importantly, both involve the same error measure 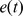. This means that a single global error signal transmitted to all network units and to the output is sufficient to guide learning. The modifications on network unit 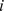 are identical to those that would be applied by FORCE learning to a sparse output unit with connections specified by 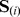. In other words, each unit of the network is being treated as if it was a sparse readout trying to reproduce, as part of its input, the desired output of the full network. The self-sensing condition, which assures that this procedure works, relies on the same incoherence and isotropy conditions as compressed sensing. These assure that units with a sufficient number of randomly selected inputs have access to all, or essentially all, of the information that they would receive from a complete set of inputs. In this sense, a sparsely connected network in a self-sensing state acts as if it was fully connected.

## Supporting Information

Appendix S1 Equations with Indices for “internal” FORCE Learning Rule.
(PDF)
