# Supplementary material for: Transferring Learning from External to Internal Weights in Echo-State Networks with Sparse Connectivity
Source: PLoS One. 2012 May 24;7(5):e37372. doi: 10.1371/journal.pone.0037372 (PMC3360031; doi:10.1371/journal.pone.0037372)
Supplement: Appendix S1 — Equations with Indices for “internal” FORCE Learning Rule. (PDF) [file pone.0037372.s001.pdf]

Transferring Learning from External to Internal Weights in Echo-State Networks with Sparse Connectivity

David Sussillo[1,*], L.F. Abbott[2]
1 David Sussillo Department of Electrical Engineering, Stanford University, Stanford, CA, USA
2 L.F. Abbott Department of Neuroscience, Columbia University, New York, NY, USA
∗ E-mail: sussillo@stanford.edu

Appendix - Equations with Indices

To avoid any ambiguities in our equations due to the rather spare matrix notation, we repeat the important equations with all the component indices intact. The notation required to do this is somewhat unwieldy, but it provides clarity and corresponds to what needs to be done to implement our results efficiently in a computer program. We introduce the indexed index K_a^i for $a=1,2,\ldots n$ to denote the n values of k for which $J_{ik}\neq 0$. Thus, $J_{iK_a^i}$ for $a=1,2,\ldots n$ denotes the weights of all the connections onto unit i and corresponds to the n components of $\mathbf{j}_{(i)}$. Similarly, the rates of the units providing this input are denoted by $r_{K_a^i}$ for $a=1,2,\ldots n$, which corresponds to $\mathbf{S}_{(i)}\mathbf{r}$. Using this notation, the equation for unit i in a sparse network without output feedback (analogous to equation 8 in the main text) is

$$\tau\frac{dx_i}{dt} = -x_i + \sum_{a=1}^{n} J_{iK_a^i} r_{K_a^i} + I_i(t)\,. \qquad (1)$$

Transfer of learning, as described in equation 21 in the main text, is expressed using this index notation as

$$\delta J_{iK_a^i} = u_i \sum_{b=1}^{n} P_{ab}^{(i)} \sum_{k=1}^{N} C_{K_b^i k} w_k\,. \qquad (2)$$

In this equation, we have labeled the components of $\mathbf{w}$ by w_k. In addition, $C_{K_b^i k}$ is the indicated element of the full rate-rate correlation matrix and $P_{ab}^{(i)}$ is defined by

$$\sum_{b=1}^{n} P_{ab}^{(i)} C_{K_b^i K_c^i} = \delta_{ac} \qquad (3)$$

for all a and c. Note that $P_{ab}^{(i)}$ is the ab component of the matrix denoted in the main text by $\mathbf{P}_{(i)}$. We have changed the subscript (i) into a superscript to make room for the matrix indices.

The learning rules corresponding to equations 22 and 23 in the main text, written with indices, are

$$P_{ab}^{(i)}(t) = P_{ab}^{(i)}(t-\Delta t) - \frac{\sum_c P_{ac}^{(i)}(t-\Delta t) r_{K_c^i}(t) \sum_d P_{bd}^{i}(t-\Delta t) r_{K_d^i}(t)}{1+\sum_{c,d} P_{cd}^{(i)}(t-\Delta t) r_{K_c^i}(t) r_{K_d^i}(t)} \qquad (4)$$

and

$$\Delta J_{iK_a^i}(t) = e(t) u_i \sum_{b=1}^{n} P_{ab}^{(i)}(t) r_{K_b^i}(t)\,. \qquad (5)$$
